# FTY720 Inhibits Expansion of Breast Cancer Stem Cells via PP2A Activation

**DOI:** 10.3390/ijms22147259

**Published:** 2021-07-06

**Authors:** Naoya Hirata, Shigeru Yamada, Shota Yanagida, Atsushi Ono, Yasunari Kanda

**Affiliations:** 1 Division of Pharmacology, National Institute of Health Sciences, Kanagawa 210-9501, Japan; n-hirata@nihs.go.jp (N.H.); shyamada@nihs.go.jp (S.Y.); S-yanagida@nihs.go.jp (S.Y.); 2 Pharmacological Evaluation Institute of Japan (PEIJ), Kanagawa 210-0821, Japan; 3 Division of Pharmaceutical Sciences, Graduated School of Medicine, Dentistry and Pharmaceutical Sciences, Okayama University, Okayama 700-8525, Japan; atsushiono3@okayama-u.ac.jp

**Keywords:** ALDH, cancer stem cells, drug repositioning, SphK1, FTY720, PP2A

## Abstract

Growing evidence suggests that breast cancer originates from a minor population of cancer cells termed cancer stem cells (CSCs), which can be identified by aldehyde dehydrogenase (ALDH) activity-based flow cytometry analysis. However, novel therapeutic drugs for the eradication of CSCs have not been discovered yet. Recently, drug repositioning, which finds new medical uses from existing drugs, has been expected to facilitate drug discovery. We have previously reported that sphingosine kinase 1 (SphK1) induced proliferation of breast CSCs. In the present study, we focused on the immunosuppressive agent FTY720 (also known as fingolimod or Gilenya), since FTY720 is known to be an inhibitor of SphK1. We found that FTY720 blocked both proliferation of ALDH-positive cells and formation of mammospheres. In addition, we showed that FTY720 reduced the expression of stem cell markers such as Oct3/4, Sox2 and Nanog via upregulation of protein phosphatase 2A (PP2A). These results suggest that FTY720 is an effective drug for breast CSCs in vitro.

## 1. Introduction

Growing evidence suggests that many types of cancer including breast cancer are initiated from a small population of cancer stem cells (CSCs) [1,2,3,4,5,6,7,8]. This minor population produces the bulk of cancers through continuous self-renewal and differentiation, which contributes to cancer heterogeneity. CSCs have been considered to have similar properties to embryonic and normal adult stem cells [9]. Thus, CSCs have been isolated from diverse tumors and established cell lines by using several methods such as stem cell markers [1], mammosphere-forming ability [3], and an activity of aldehyde dehydrogenase (ALDH), a detoxifying enzyme responsible for the oxidation of intracellular aldehydes [4]. Since CSCs are considered to have the abilities of a drug resistance and tumor recurrence initiation, novel therapeutic drugs for the eradication of CSCs have been required for cancer treatment.

In general, drug discovery for cancer therapy has involved costly and time-consuming processes, with a low probability of clinical trials. Recently, there has been increased focus on drug repositioning (DR), which is an idea to use previously approved drugs for a different disease away from their original intended use [10,11,12]. These repositioned drugs have already been studied for their pharmacokinetics, medical efficacy, and safety. Thus, this approach can be expected to save time and money in drug development, and further accelerate their entry to clinical trials.

We have demonstrated that a bioactive lipid mediator sphingosine-1-phosphate (S1P) regulates expansion of breast CSCs [13]. Our findings indicate that sphingosine kinase 1 (SphK1), an S1P-producing enzyme, regulates the proliferation and tumorigenicity of breast CSCs. Considering these target-based DR approaches, we focused on a SphK1 inhibitor FTY720 [14], which is used for multiple sclerosis treatment as an immunosuppressive agent [15].

In the present study, we investigated the effect of FTY720 on the proliferation of breast CSCs using both luminal breast cancer cell line MCF-7 and triple-negative breast cancer cell line MDA-MB-231. We found that FTY720 inhibits the expansion of breast CSCs along with stem cell marker suppression. We further showed that PP2A mediates the inhibition of breast CSC expansion by FTY720. Thus, these results suggest the potential effectiveness of FTY720 as a therapeutic agent for breast cancer.

## 2. Results

### 2.1. Growth Inhibition of Breast CSCs by FTY720

**Figure 1:**
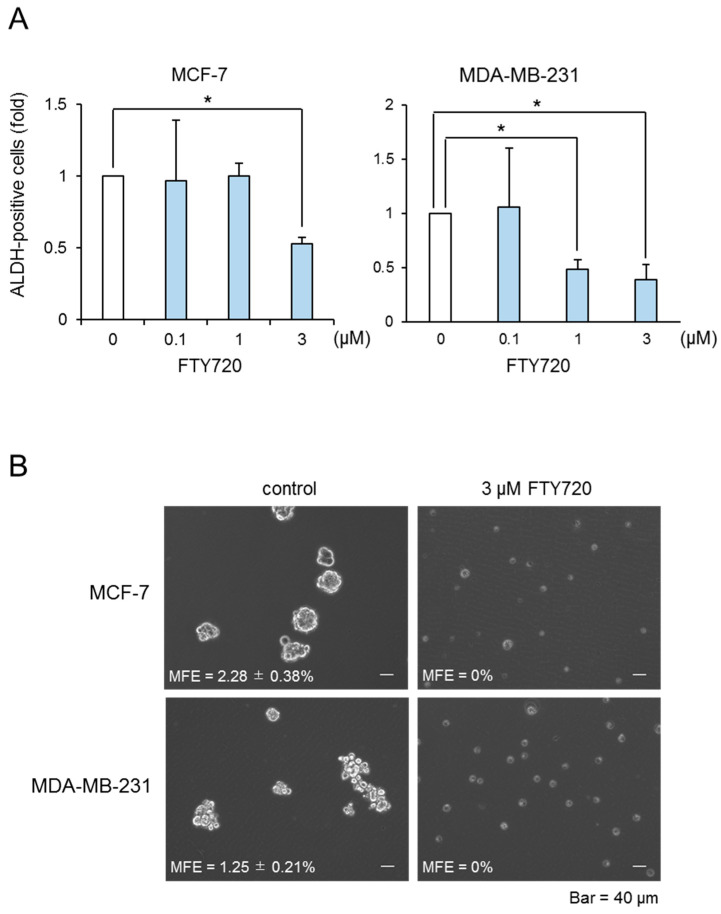
Effect of FTY720 on breast CSC proliferation. (**A**) After treatment with different concentration of FTY720 for 3 days, the ALDH-positive cells of MCF-7 cells (**left**) and MDA-MB-231 cells (**right**) were assessed using the ALDEFLUOR kit and flow cytometry. (**B**) Effect of FTY720 (3 µM) on mammosphere-forming efficiency in MCF-7 and MDA-MB-231 cells. The number of mammospheres was microscopically counted and the percentage of mammosphere-forming cells was determined as mammosphere-forming efficiency (MFE; %). The scale bar indicates 40 µm. Data represent mean ± s.d. (*n* = 3). * *p* < 0.05.

We performed the DR approach to find CSC-targeted drugs. Since we have previously reported that SphK1 induced proliferation of breast CSCs [13], we focused on the immunosuppressive agent FTY720, which is well known to be a SphK1 inhibitor and is used as immunosuppressive agents clinically. In our previous report, we demonstrated that ALDH-positive cells in breast cancer cells possess CSC-like properties, as assessed by the expression of stem cell markers, drug resistance, and tumorigenicity [13]. Here, we investigated the effect of FTY720 on the proliferation of breast ALDH-positive cells from the MCF-7 cell line (luminal type) and MDA-MB-231 cell line (triple-negative type). As shown in Figure 1A, FTY720 decreased the proportion of ALDH-positive cells from both cell lines in a dose-dependent manner, with a maximal effect observed at 3 µM. FTY720 decreased the cell number of ALDH-positive and ALDH-negative cell populations and exhibited selective cytotoxicity against ALDH-positive cells (Table S1). In contrast, anti-cancer agents such as 5-fluorouracil (5-FU) and doxorubicin (Dox) did not have suppressive effects on these CSCs (Figure S1). To confirm these observations, we examined the effect of FTY720 on breast CSCs using a mammosphere-forming assay, which is widely used to assess the self-renewal capacity of CSCs [13]. FTY720 inhibited mammosphere formation in both cell lines (Figure 1B). These results suggest that FTY720 is a potential effective drug for breast CSCs.

### 2.2. Downregulation of Stem Cell Marker Genes by FTY720

**Figure 2:**
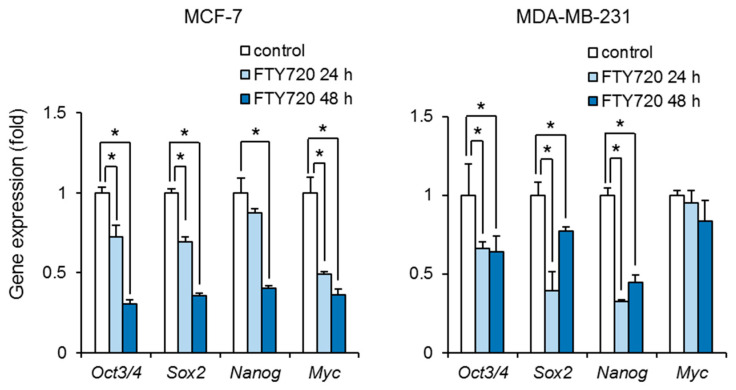
Effect of FTY720 on stem cell marker genes in breast CSCs. After treatment with FTY720 (3 µM) for 24 and 48 h, expression levels of stem cell marker genes in MCF-7 cells (**left**) and MDA-MB-231 cells (**right**) were measured by real-time RT-PCR. Data represent mean ± s.d. (*n* = 3). * *p* < 0.05.

We next examined the effect of FTY720 on the stemness of breast CSCs. Treatment with FTY720 significantly decreased the expression of Oct3/4, Sox2, Nanog, and Myc in ALDH-positive cells in MCF-7 cells (Figure 2). FTY720 also decreased the expression of Oct3/4, Sox2, and Nanog in ALDH-positive cells in MDA-MB-231 cells (Figure 2). These results suggest that FTY720 inhibits the stemness in breast CSCs.

### 2.3. Upregulation of PP2A Activity by FTY720

**Figure 3:**
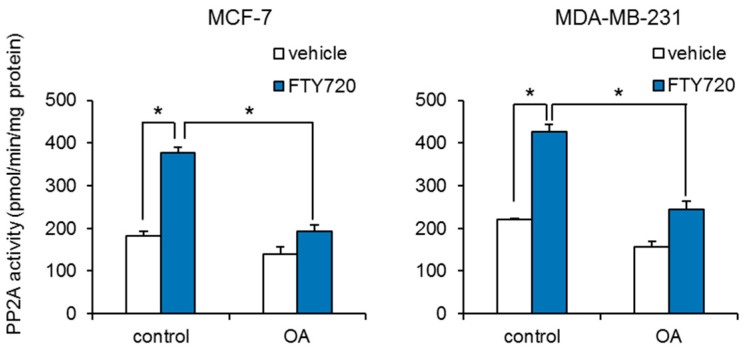
Effect of FTY720 on PP2A activity in breast cancer cell lines. After treatment with FTY720 (3 µM) and/or PP2A inhibitor okadaic acid (OA, 30 nM) for 24 h, PP2A activities of MCF-7 cells (**left**) and MDA-MB-231 cells (**right**) were measured. Data represent mean ± s.d. (*n* = 3). * *p* < 0.05.

We further investigated the mechanism by which FTY720 decreases breast CSCs. It has been reported that FTY720 induces cell cycle arrest and apoptosis via increase in the expression of tumor suppressor p53 [16]. We examined whether FTY720 induced the expression of p53. FTY720 has no effect on the expression of p53 in both MCF-7 and MDA-MB-231 cells (Figure S2). We next studied whether protein phosphatase 2A (PP2A) is involved in the FTY720-induced growth inhibition of breast CSCs, since PP2A is known to inhibit SphK1 through its dephosphorylation [17], and FTY720 activates PP2A through interaction with SET, which is a regulator of PP2A activity [18]. We found that FTY720 significantly increased the PP2A enzyme activities (Figure 3). In addition, FTY720-induced PP2A activities were abolished by treatment with the PP2A selective inhibitor okadaic acid (Figure 3). Treatment with okadaic acid alone had little effect on PP2A activities in both cells. These data suggest that FTY720 upregulates PP2A enzyme activity in breast cancer cells.

### 2.4. Effect of PP2A Inhibitor on FTY720-Induced Negative Regulation

**Figure 4:**
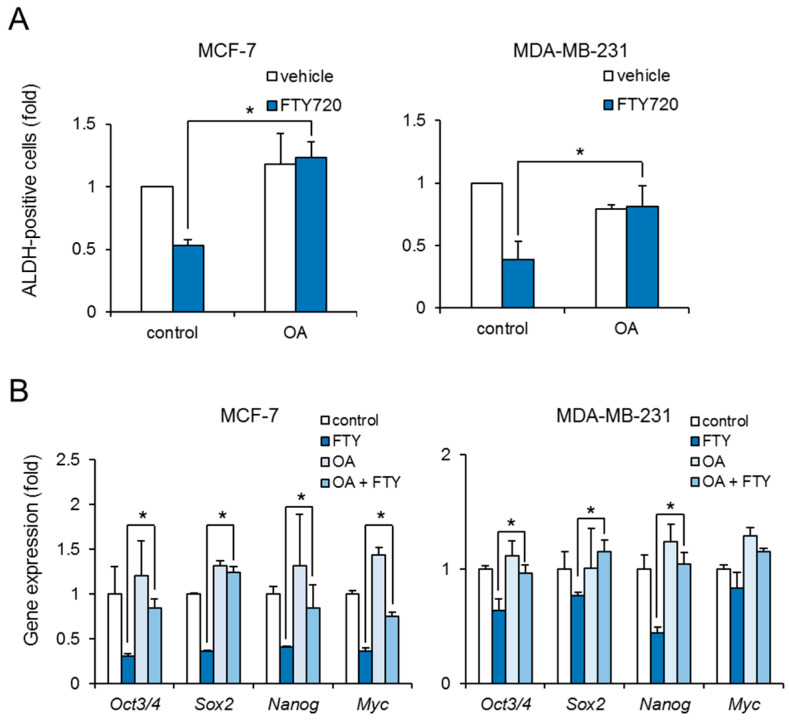
Effect of PP2A inhibitor on FTY720-induced negative regulation in breast CSCs. (**A**) After treatment with FTY720 (3 µM) and/or OA (30 nM) for 3 days, the ALDH-positive cells of MCF-7 cells (**left**) and MDA-MB-231 cells (**right**) were assessed using the ALDEFLUOR kit and flow cytometry. (**B**) After treatment with FTY720 (3 µM) and/or OA (30 nM) for 48 h, expression levels of stem cell marker genes in MCF-7 cells (**left**) and MDA-MB-231 cells (**right**) were measured by real-time RT-PCR. Data represent mean ± s.d. (*n* = 3). * *p* < 0.05.

To reveal whether FTY720-induced growth inhibition of breast CSCs was mediated through PP2A activation, we examined the effect of okadaic acid on ALDH-positive cells. Treatment with okadaic acid abolished the reduction of ALDH-positive cells by FTY720 in both cell lines (Figure 4A). Moreover, okadaic acid recovered the FTY720-induced downregulation of stem cell markers at 48 h (Figure 4B). These results suggest that FTY720 negatively regulates the stemness and growth of breast CSCs via PP2A activation.

## 3. Discussion

In the present study, we demonstrated that FTY720 decreased the breast CSC population in both MCF-7 and MDA-MB-231 cell lines. We also found that FTY720-induced reduction of breast CSCs is mediated by PP2A. These findings suggest an effectiveness of FTY720 against breast cancer cells.

We found that FTY720 at a few µM suppressed the breast CSC expansion (Figure 1). Cancer stem cells are known to be resistant to chemotherapy because of elevated expression of drug-efflux pumps such as ABC transporters [19]. Indeed, we found that anti-cancer agents 5-FU and Dox did not have suppressive effects on breast CSCs. Since FTY720 showed selective cytotoxic effects on CSCs, combination with conventional anti-cancer agents such as 5-FU and doxorubicin might be useful for cancer therapy. In addition, it has been reported that the plasma FTY720 concentration in patients with atherosclerosis was detected at micromolar levels [20]. Our in vitro observations with FTY720 were within a similar range of concentrations and are expected to be effective in cancer treatment.

The CSC population of MDA-MB-231 cells was more effectively reduced by FTY720 than that of MCF-7 cells (Figure 1A). Along with this, Sox2 and Nanog expressions of MDA-MB-231 cells were more potently downregulated after FTY720 treatment for 24 h than those of MCF-7 cells (Figure 2). Since CSCs produces the bulk of cancers through continuous self-renewal and differentiation, which contributes to cancer heterogeneity and malignancies such as recurrence, metastatic potential, and drug resistance, it is essential to target CSC regulators for cancer treatment [21]. Both Sox2 and Nanog are known to be involved in breast CSC regulation including growth [22,23]. In addition, we previously reported that MDA-MB-231 cells highly contained CSCs than MCF-7 cells [13]. Thus, the repressive effect of FTY720 on highly malignant breast cancer might be triggered by downregulation of these CSC factors.

Our data showed that FTY720-induced breast CSC reduction is mediated by PP2A activation (Figure 3 and Figure 4). PP2A activation is known to decrease phosphorylation of ERK and inactivate it [24]. Moreover, FTY720 has been reported to inactivate ERK and decrease brain tumor stem cells (BTSCs) derived from human glioma tissue [25]. The mechanism of FTY720-induced BTSC reduction has been shown to be through activation of the intrinsic mitochondrial pathway, which is evidenced by the rapid accumulation of Bim via ERK inactivation, leading to caspase-9 and eventually caspase-7 or caspase-3 activation [25]. Thus, FTY720-induced growth inhibition of breast CSCs might also be mediated by the mitochondrial pathway like BTSCs.

FTY720 has been reported to be phosphorylated by SphK2 and released from cells to bind S1PR1, which results in functional antagonism by inducing receptor internalization and proteasomal degradation in immune cells [26]. Thus, chronic exposure to FTY720 is thought to induce a decrease in S1PR1 levels, thereby reducing inflammatory immune response. Despite increasing concerns, antagonism of FTY720 on other S1PRs (S1PR3-5) has not been elucidated. Among S1PRs, S1PR3 is known to be most highly expressed in breast cancer cells [27]. In addition, we previously indicated that S1PR3 plays a major role in S1P-induced proliferation of breast CSCs [13]. Further research is required to determine whether FTY720 also targets and desensitizes S1PR3 by its degradation in breast CSCs.

FTY720, an immunomodulator drug, are known to inhibit the NF-κB/IL-6/STAT3 signaling cascade. Breast CSCs are considered to be surrounded in the tumor microenvironment and are regulated by proinflammatory cytokines from immune cells [28]. Recently, inhibition of the NFκB-STAT3 signaling pathway by FTY720 has been reported to prevent the production of inflammatory cytokines and suppress the progression of breast cancer [29]. Further study should be performed to elucidate whether FTY720 inhibits breast cancer via both direct effects on CSCs and indirect effects by inhibiting cytokine production, which may facilitate breast cancer progression.

In summary, our results demonstrate a success case of DR for breast cancer treatment by targeting breast CSC population. We could first determine FTY720 as an effective drug against breast CSC regulation by DR. Further research is needed to evaluate the proliferative effects of FTY720 on other CSC types.

## 4. Materials and Methods

### 4.1. Chemicals

FTY720 was from Sigma-Aldrich (St. Louis, MO, USA). Okadaic acid was from Wako Pure Chemical (Tokyo, Japan). 5-Fluorouracil and doxorubicin were from Enzo Life Sciences (Farmingdale, NY, USA). All other reagents were of analytical grade and obtained from commercial sources.

### 4.2. Cell Culture

MCF-7 and MDA-MB-231 cells (American Type Culture Collection, Manassas, VA, USA) were cultured in Dulbecco’s modified Eagle’s medium (DMEM; Sigma-Aldrich) supplemented with 10% heat-inactivated fetal bovine serum (FBS; Biological Industries, Ashrat, Israel), 100 U/mL penicillin, and 100 µg/mL streptomycin (Thermo Fisher Scientific, Waltham, MA, USA).

### 4.3. ALDH Assays

The ALDEFLUOR kit (Stem Cell Technologies, Vancouver, BC, Canada) was used to detect CSC populations with high ALDH enzyme activity according to the manufacturer’s instruction [13,30,31]. The cells were plated at a density of 1 × 10^5^ cells in 60 mm culture dishes. After serum deprivation for 3 days, cells were suspended at a concentration of 1 × 10^6^ cells/mL in ALDH assay buffer containing the ALDH substrate BAAA (1 µM) and incubated for 30 min at 37 °C. As a negative control, cells were treated with diethylaminobenzaldehyde (DEAB, 15 µM), a specific ALDH inhibitor. A FACS Aria II cell sorter (BD Biosciences, San Jose, CA, USA) was used to measure the ALDH-positive cells.

### 4.4. Mammosphere-Forming Assays

The cells were plated as single cells on ultra-low attachment 6-well plates (Corning, Inc., Corning, NY, USA) at a concentration of 10,000 cells/mL in serum-free DMEM supplemented with N_2_ supplement (Thermo Fisher Scientific) and 20 ng/mL basic Fibroblast Growth Factor (R&D Systems, Minneapolis, MN, USA). After 4 days, the number of mammospheres was microscopically counted and the percentage of mammosphere-forming cells was determined as mammosphere-forming efficiency (MFE; %) [5,13].

### 4.5. Real-Time Polymerase Chain Reaction (PCR)

Total RNA was isolated from the cells using Trizol (Thermo Fisher Scientific), according to the manufacturer’s instructions. The qPCR assays were conducted with the aid of a QuantiTect SYBR Green RT-PCR Kit (QIAGEN, Valencia, CA, USA) and an ABI PRISM 7900HT sequence detection system (Applied Biosystems, Foster City, CA, USA) as previously described [32]. The relative changes in transcript levels for each sample were determined by normalizing to GAPDH mRNA levels. The following primer sequences were used for real-time PCR analysis: Oct3/4, forward, 5′-ACATCAAAGCTCTGCAGAAAGAA-3′ and reverse, 5′- -3′; Sox2, forward, 5′-AACCCCAAGATGCACAACTC-3′ and reverse, 5′-GCTTAGCCTCGTCGATGAAC-3′; Nanog, forward, 5′-CAGAAGGCCTCAGCACCTAC-3′ and reverse, 5′-ATTGTTCCAGGTCTGGTTGC-3′; Myc, forward, 5′-CACGAAACTTTGCCCATAGC-3′ and reverse, 5′-GCAAGGAGAGCCTTTCAGAG-3′; GAPDH, forward, 5′-GTCTCCTCTGACTTCAACAGCG-3′ and reverse, 5′-ACCACCCTGTTGCTGTAGCCAA-3′.

### 4.6. PP2A Activity

PP2A activity was measured using a PP2A Immunoprecipitation Phosphatase Assay Kit according to the manufacturer’s instruction. The cells were lysed with a single freeze–thaw cycle in 50 mM Tris HCl, pH 7.4, 150 mM NaCl, 1 mM EDTA, 0.1% Triton X-100, and complete Mini EDTA-free Protease Inhibitor Cocktail (Roche, Basel, Switzerland). Lysed cells were centrifuged at 20,000 g for 15 min and the supernatant was collected. Approximately 200 µg of proteins was mixed with anti-PP2A antibody and Protein A agarose. After rotation at 4 °C for 2 h, samples were washed with TBS and assay buffer. Samples were resuspended with assay buffer containing phosphopeptide and incubated at 30 °C for 10 min. Malachite Green Solution was added and the absorbance at 630 nm was measured using an iMark Microplate Reader (Bio-Rad, Hercules, CA, USA). The amount of phosphate was calculated from a standard curve and normalized to the total protein content.

### 4.7. Statistical Analysis

Results are shown as mean ± s.d. Statistical analyses were performed using Excel 2010. *p*-values were calculated using a two-sided unpaired Student’s *t*-test. Differences at *p* < 0.05 were considered to be significant.

## Data Availability

No data Available.

## Supplementary Materials

The following are available online at https://www.mdpi.com/article/10.3390/ijms22147259/s1, Table S1: Cell viability of FTY720-treated cells. Figure S1: Comparison of FTY720 with anti-cancer drug 5-FU and doxorubicin. Figure S2: Effect of FTY720 on tumor suppressor p53 expression.
