# Supplementary material for: FTY720 Inhibits Expansion of Breast Cancer Stem Cells via PP2A Activation
Source: Int J Mol Sci. 2021 Jul 6;22(14):7259. doi: 10.3390/ijms22147259 (PMC8329924; doi:10.3390/ijms22147259)
Supplement: Supplementary file 1 [file ijms-22-07259-s001.zip › ijms-1267504-SI.pdf]

Table S1. Cell viability of FTY720-treated cells. After treatment with FTY720 for 72 h, number of the ALDH-negative and -positive cells in MCF-7 cells (left) and MDA-MB-231 cells (right) were assessed using the Aldefluor assay kit and flow cytometry. Cell viability was indicated as percent of control. Data represent mean ± s.d. (n = 3).

cell line / μM	MCF-7		MDA-MB-231	
	ALDH (-)	ALDH (+)	ALDH (-)	ALDH (+)
0.1	88.2±5.2	83.5±31.0	93.2±5.9	99.7±56.9
1	72.5±4.0	69.3±8.7	72.9±9.9	34.0±6.0
3	64.3±3.4	35.2±4.9	59.4±2.9	21.6±8.0

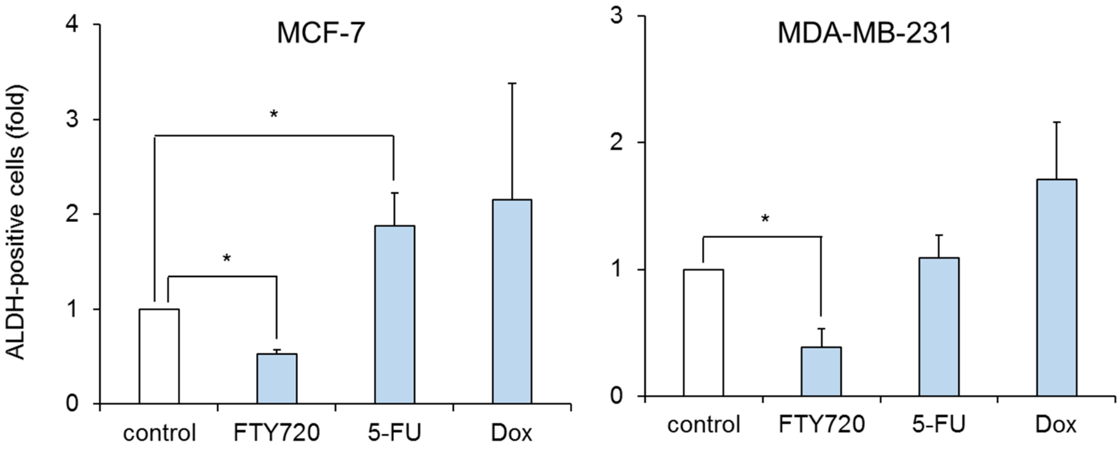

Figure S1. Comparison of FTY720 with anti-cancer drug 5-FU and doxorubicin. After treatment with FTY720 (3 M), 5-FU (10 M) or Dox (1 M) for 72 h, the ALDH-positive cells of MCF-7 cells (left) and MDA-MB-231 cells (right) were assessed using the Aldefluor assay kit and flow cytometry. Data represent mean ± s.d. (n = 3). * $p < 0.05$.

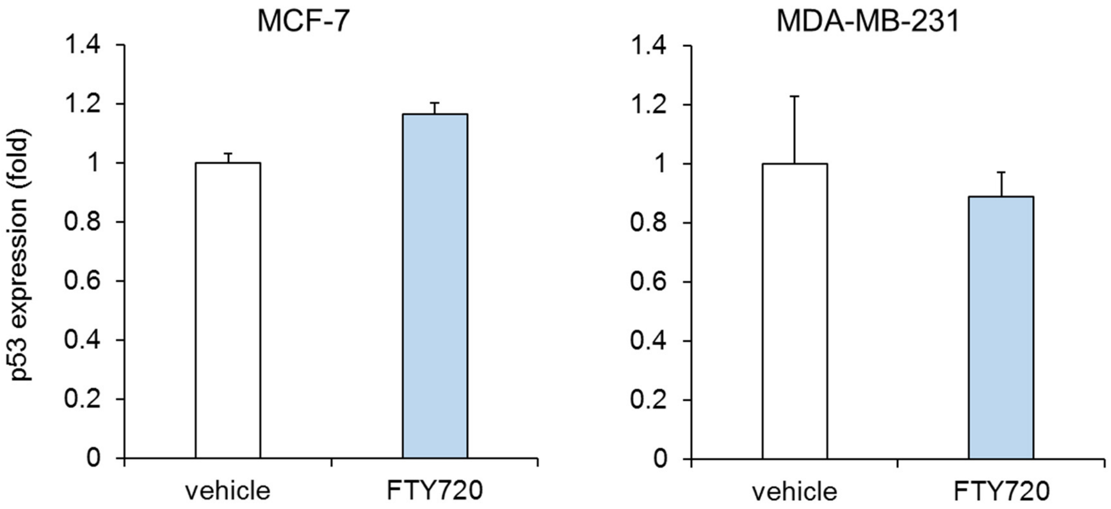

Figure S2. Effect of FTY720 on tumor suppressor p53 expression. After treatment with FTY720 (3 M) for 48 h, expression level of p53 in MCF-7 cells (left) and MDA-MB-231 cells (right) were measured by real-time RT-PCR. Data represent mean ± s.d. (n = 3).
